# Crows control working memory before and after stimulus encoding

**DOI:** 10.1038/s41598-020-59975-4

**Published:** 2020-02-24

**Authors:** Erica Fongaro, Jonas Rose

**Affiliations:** Faculty of Psychology, Neural Basis of Learning, Ruhr University Bochum, 44801 Bochum, Germany

**Keywords:** Cognitive control, Working memory, Animal behaviour

## Abstract

The capacity of working memory is limited and this limit is comparable in crows and primates. To maximize this resource, humans use attention to select only relevant information for maintenance. Interestingly, attention-cues are effective not only before but also after the presentation of to-be-remembered stimuli, highlighting control mechanisms beyond sensory selection. Here we explore if crows are also capable of these forms of control over working memory. Two crows (*Corvus corone*) were trained to memorize two, four or six visual stimuli. Comparable to our previous results, the crows showed a decrease in performance with increasing working memory load. Using attention cues, we indicated the critical stimulus on a given trial. These cues were either presented before (pre-cue) or after sample-presentation (retro-cue). On other trials no cue was given as to which stimulus was critical. We found that both pre- and retro-cues enhance the performance of the birds. These results show that crows, like humans, can utilize attention to select relevant stimuli for maintenance in working memory. Importantly, crows can also utilize cues to make the most of their working memory capacity even after the stimuli are already held in working memory. This strongly implies that crows can engage in efficient control over working memory.

## Introduction

Working memory (WM) refers to the ability to memorize information over short periods and, importantly, to process this information. This makes WM a core component of cognition that is critical for decision making, planning, goal directed behavior and many other cognitive abilities^1,2^. It is well established that WM is not a passive storage but that it requires active maintenance of information^3^. Consequentially, WM is not only vulnerable to distraction but also limited in duration and in capacity^4–6^. It was estimated that healthy humans can maintain about three to five items in working memory^7–11^. Capacity-limitation is a critical bottleneck of cognition and has recently been coined the ‘bandwidth of cognition’^12,13^.

In light of these limitations, it is evident that WM must rely on effective top-down control^14^. This control can occur in different ways. Attention can act as a ‘gatekeeper’ that prioritizes the encoding of relevant sensory stimuli and filters out irrelevant information^14–18^. While this mechanism is highly effective, it requires prior knowledge about the relevance of upcoming sensory events. If this information is not available at the time of encoding, the system also needs to control the maintenance of information that is already held in WM. In humans, both mechanisms are well established - attention is used to gate stimuli for WM and executive control selects from information already maintained in WM. Pioneering work from Posner and Cohen^19^ demonstrated that visual cues facilitate target detection if a location is cued prior to the presentation of the target. Likewise, such ‘pre-cues’ facilitate the retrieval of visual stimuli following a period of WM maintenance^15,16,20–24^. This illustrates the importance of attentional ‘gatekeeping’ for visual WM. Most interestingly, cue-stimuli are effective also *after* stimulus-presentation. Like pre-cues, such ‘retro-cues’ increase the retention of cued material albeit to a lesser extent^25–33^. Since this effect cannot be driven by sensory gate-keeping it must rely on some form of control mechanism that acts on information that is already maintained in WM.

Outside the context of WM many species can use cues to direct attention to relevant stimuli. This was, for instance, demonstrated in monkeys^34–36^, rats^37,38^, chickens^39^ and pigeons^40,41^. The physiological mechanism of WM, active maintenance, also appears to be conserved across species^42–53^ (even though the latest models have not yet been tested^54^). Therefore, it seems likely that any species with a well-developed cognitive skillset should be able to gate WM using predictive cues. Clear evidence, however, has so far only been reported in primates^28,55^.

The question if animals can improve WM performance *after* the stimuli have been encoded has received only little attention. To the best of our knowledge, only one study examined this effect in rhesus monkeys^56^. In the study, arrays of two or three visual stimuli were presented followed by a retro-cue that indicated relevant stimuli after stimulus-presentation. The monkeys showed some benefit from the cue at a load of two stimuli but not at a load of three stimuli. The authors concluded that a memory load of three images could result in “insufficient memory strength” for the retro-cue to have an effect^56^. A different protocol, directed forgetting, was used with a similar aim – to investigate the executive control over WM in animals^51,57,58^. In directed forgetting paradigms, cues instruct the subjects if a memorized stimulus will later be tested or if a test is omitted. While such ‘forget-cues’ result in a dramatic decline in recognition, it is not fully resolved if this reflects executive control or if the results can be explained by simpler mechanisms such as motivational differences^59,60^. This is a problematic notion since forgetting can only be tested on few probe trials where the animals do not expect a test and do not have a chance to receive a reward for a correct choice. If the procedure is modified such that the animals will receive a ‘free’ reward following the cue to forget then forgetting is not evident^61^. Furthermore, a directed forgetting procedure cannot directly asses the modulation of WM capacity.

Here we test the control over WM in crows, animals with a large cognitive repertoire that some compare to that of apes^62^. For instance, some corvids manufacture and use tools^63^, exhibit episodic-like memory^62^, master elaborate tests of object permanence^64^ and show impressive problem-solving abilities^65–69^. While these abilities suggest that corvids should be able to direct attention to relevant stimuli and control their cognitive resources, it was never directly demonstrated. We recently showed that crows have a high WM capacity that is comparable to the WM capacity of rhesus monkeys in a virtually identical paradigm^5,70^. Here, we modified the paradigm and included trials with pre- or retro-cues while the animals performed either below, at, or above their WM capacity. The modification allowed us to test whether crows can optimize WM capacity using attentional gating to select relevant information during stimulus encoding. Importantly, the design also allowed to test if crows can control WM and select among already maintained stimuli after the encoding of the stimulus material. Such a retro-cue effect would indicate that crows show some form of executive control over WM.

## Materials and Methods

### Subjects

Two hand raised male carrion crows (*Corvus corone*), 3 years of age, with baseline weights of 505 g and 500 g, were used. The crows were housed in a social group of 5 birds in an indoor aviary with a controlled 12 hours day/night cycle. When not in experiments, the birds were given ad libitum access to water, grit and food (Versele Laga Nutribird F16, BeoSoft, occasionally nuts, fruit, chicks and mealworms). During experiments the crows were maintained on a controlled food protocol with free access to water and grit, such that food-pellets (Nutribird F16, Versele-Laga, Germany) could be used as a reward during each training session in the operant-chamber. The animals’ body weight was controlled daily and maintained between 85% and 95% of their free feeding weight. All preparations and procedures were performed according to the principles of the care and use of laboratory-animals adopted by the German Animal Welfare Law for the prevention of cruelty to animals and were conducted after approval by the LANUV (Landesamt für Natur, Umwelt und Verbraucherschutz Nordrhein-Westfalen).

### Experimental setup

The setup consisted of a chamber (50 cm wide × 50.5 cm deep × 77.5 cm high), equipped with a remote monitoring camera (Sygonix, Taiwan), a 22” touchscreen-monitor (ELO 2200 L APR, Elo Touch Solutions Inc., USA) and a custom-made automatic pellet feeder (plans available: www.jonasrose.net). The bird was placed on a wooden perch in front of the touchscreen such that the maximum distance from the bird’s eye to the screen was 7 cm. The animal’s head position and rotation was tracked in the horizontal plane, using a computer-vision camera (Chameleon3, Point Grey Research Inc., Canada) with a frame rate of 150 Hz. For head-tracking (Supplementary Fig. S1), a custom 3d-printed reflector was mounted on a surgically implanted, light-weight (<350 mg), custom head-post and removed after each experimental session. This tracking allowed to train the animals to hold the head still and straight throughout the paradigm – important to prevent non-cognitive strategies. All experiments were controlled by custom programs in Matlab (Mathworks Inc. Natick, MA USA) using the Biopsychology^71^ and Psychophysics toolboxes^72^. Digital input and output of the control PC was handled by a microcontroller (ODROID C1, Hardkernel co. Ltd) running custom software (available: www.jonasrose.net) connected through gigabit network. This allowed for flexible and temporally precise IO-communication.

### Behavioral protocol

**Figure 1:**
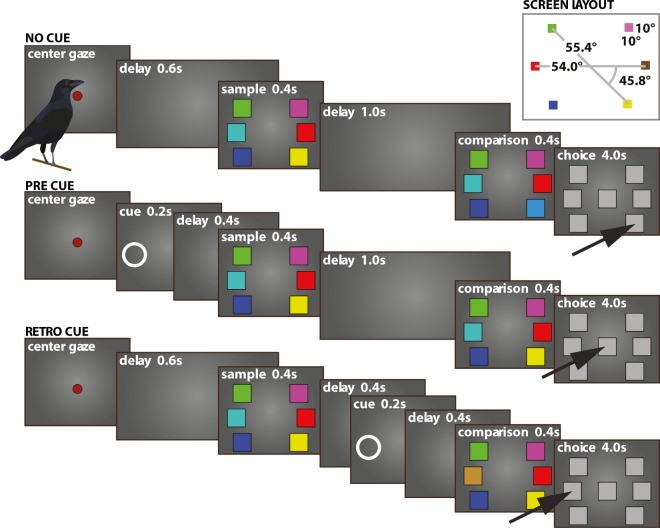
Behavioral protocol: The change detection task. Following the presentation of a red dot in the center of the screen the crows had to hold the head straight and centered to start the trial. They had to maintain this head-position until the choice period at the end of the trial. Each training session consisted of no-cue, pre-cue and retro-cue conditions. The overall duration of the conditions was matched. In all conditions the animals viewed an array of 2, 4 or 6 colored squares for 0.4 s. After a memory delay of 1.0 s the array re-appeared for 0.4 s. On 50% of the trials one color was exchanged and the animals had to indicate during the choice period if any color was changed in the new array. A peck to the center indicated no change while a peck on any of the peripheral stimuli indicated change. In the pre- and retro-cue conditions an additional stimulus was presented to cue the location at which the color-change would occur. If no change occurred the cue-location was chosen at random. On pre-cue trials the cue was presented before the sample-array while on retro-cue trials it was presented following the sample-array during the memory-delay.

The crows were previously trained and tested on a change localization paradigm^70^ beginning 2 years prior to the present study. They were then retrained for approximately 4 months on a change detection paradigm (Fig. 1). Following the transition to the change-detection procedure the animals learned the cued change detection paradigm, first in an unpublished project without head-tracking, then they were introduced to the full paradigm reported in this study. Following the training period, the animals were tested and data were collected over ten consecutive testing sessions. On each training and testing session, the animals performed 850 trials (approximately 190 min) interrupted by brief breaks with access to water.

Before each training session, the animals voluntarily flew to the extended arm of the experimenter who then transported the animal to the operant chamber. Each trial started with an inter-trial interval (4000 ms) after which the crows were presented with a red dot in the center of the screen (maximal 5000 ms) that served as a cue to start the trial. We used a head-tracking system to monitor head-movements of the crows. A trial started when the crows centered their heads and looked straight at the red dot (±2 cm horizontal displacement, ±17° horizontal head-rotation). This head position had to be maintained until the end of the trial or the trial was aborted and a gaze-break was recorded. By training the animals to hold the head still and straight throughout the trial we were able to prevent non-cognitive strategies such as positioning the head on a cued stimulus location rather than controlling WM. This was a critical control to test attentional and executive control over WM. If a trial was not started or aborted the crows received negative feedback in the form of a timeout (9000 ms) and a brief white illumination of the screen.

After starting a trial (600 ms delay), the birds were presented with a sample-array (400 ms) that consisted of 2, 4 or 6 differently colored squares (see further details on the colors used below). On half of the trials, the sample-array re-appeared (after 1000 ms) on the other half of the trials it re-appeared (after 1000 ms) with one color exchanged. Following the presentation of this comparison-array (400 ms) all colors turned gray and one additional gray square appeared in the center of the screen. The crows were trained to indicate if the sample- and comparison-arrays were identical (a choice had to be made within 4000 ms). If there was no color-change, the birds had to peck the new square in the center. If there was a color-change at any location, the crows had to peck on any of the peripheral squares. In order to perform this paradigm successfully, the crows had to memorize the sample-array on each trial and to compare it to the comparison-array. If the animals made a correct response, a single food-pellet was delivered as reward from the automated feeding-trough, which was illuminated during reward-delivery (2000 ms). An incorrect response or a response-omission resulted in negative feedback, a brief white illumination of the screen and a time-out (9000 ms).

Each training session consisted of three conditions: a no-cue condition, a pre-cue condition and a retro-cue cue condition. While the overall trial-length of all three conditions was matched, the trials differed in the delays before or after the presentation of the sample-array. In the cue conditions, a white circle appeared for 200 ms in one of the respective test locations. This cue could appear either during the first 200 ms of the delay before the sample-array (pre-cue, followed by a 400 ms delay) or during the central 200 ms of the delay before the comparison-array (retro-cue, preceded and followed by a 400 ms delay). The cues instructed the animals that, if it was a trial with a color-change, it would occur on the cued-location. Therefore, the cues could be used either to direct attention to a specific spatial location during viewing of the sample-array (pre-cue) or to select one color from WM (retro-cue). The overall duration of the conditions was matched such that the cues did not alter the duration of the trial or the delays. On no-cue trials a sample-array-size of 2, 4 or 6 colors was used, while on pre- and retro-cue trials only arrays of size 4 and 6 were used since these were at or above the animals’ WM capacity.

The design of the stimuli was based on the protocol by Buschman *et al*.^5^. On each trial, two, four or six colors were presented at fixed screen locations. For every day, random color-combinations were chosen from a set of 14 colors such that six pairs, one for each stimulus-location were chosen on a given day. Thus, on each training day, one random pair of colors was used at each of the six stimulus locations. The order of presentation of the colors within a pair was randomized and balanced across conditions. The target location for cued and un-cued conditions and the total number of stimuli in the array and the side of the change were pseudo-randomized such that all conditions had equal likelihood on a given trial. Color-stimuli were square, 10 degrees of visual angle (DVA) on either side and placed either on the horizontal meridian of the screen or 45.8 DVA above/below the meridian at a distance from the center of 54 and 55.4 DVA (center of the stimulus) respectively^70^ (Fig. 1). The cue stimuli were white thin transparent circles (11 DVA diameter), positioned on the exact location of the color-stimuli to cue. The maximal binocular overlap for carrion crows is around 37.6 DVA^73^; thus, all color-stimuli and cues were placed outside the binocular area, taking into account head-movement, head-rotation and eye movement.

### Surgery

All surgeries were performed under aseptic conditions as part of a previous experiment^70^. For the head-tracking system, a light-weight (<350 mg) custom head-post was chronically implanted to attach a small reflector during behavioral experiments. Anesthesia was induced and maintained by ketamine (50 mg/kg) and xylazine (5 mg/kg). Once deeply anaesthetized, the crows were placed in a stereotaxic frame. Some feathers were plucked over the base of the beak exposing skin for a small incision to retract the skin. A small opening was drilled in the surface of the bone in order to expose the *trabeculae* to which the head-post was attached with dental acrylic. The inner layer of the bone remained undisturbed. The wound-margins were sutured. Following the administration of analgesia (morphasol, 3 ml/kg), the crow was placed in a recovery-cage until fully recovered (standing on a perch, eating and drinking, recovery could up to 4 hours after the procedure). The analgesia was administered for 2 days after the surgery.

### Data analysis

Data was analyzed with Matlab (Mathworks Inc. Natick, MA) using custom code and the statistics toolbox. The effect of memory load was tested in no-cue trials using one-way ANOVA with load as independent variable across days. The effect of cue-type and load were assessed with two-way ANOVA with memory load and cue-type as independent variables across days, followed by Tukey-Kramer Honest Significantly Different (HSD) post-hoc analysis. All ANOVAs were conducted for each crow independently. The effects of cue-type, load and their interaction were tested independently for each animal, using two-way ANOVA. Here several behavioral measures were used: Percent correct, percent trials with gaze-break, reaction time, sensitivity score (d’) for change/no-change discrimination (see below) and Pashler-K as unit of working-memory capacity (see below). We chose this wide array of measures to ensure comparability with different studies that often report one or the other. Analyses were based on attempted trials excluding trials with gaze-breaks (apart from the analysis of gaze-breaks).

We also analyzed the entire set of raw-data using a generalized linear mixed model (GLMM, in Matlab called GLME). The advantage of this approach was that we were able to model binary data (correct/incorrect) on each trial using a binomial distribution with logit link function. Bird served as a random variable. We used GLMM to directly examine the effect of number of items on performance and performed a model comparison (likelihood ratio test) between a full model and models that either did not include the factors pre- or retro-cue.

The sensitivity score d’ was calculated for change/no-change discrimination^74,75^ using the following equation:

$${\rm{d}}{\rm{{\prime} }}={\rm{Z}}\,({\rm{h}}{\rm{i}}{\rm{t}}\,{\rm{r}}{\rm{a}}{\rm{t}}{\rm{e}})\,-\,{\rm{Z}}\,({\rm{f}}{\rm{a}}{\rm{l}}{\rm{s}}{\rm{e}}\,{\rm{a}}{\rm{l}}{\rm{a}}{\rm{r}}{\rm{m}}\,{\rm{r}}{\rm{a}}{\rm{t}}{\rm{e}}).$$

here hit-rate was defined as the conditional probability that the participants responded “change-present” given that the change was presented, and the false-alarm rate was defined as the conditional probability that the participants responded “change-present” when the change was absent.

The WM capacity K was estimated using Pashler-K using the following equation:

$${\hat{k}}_{p}=N(\frac{\hat{h}-\hat{f}}{1-\hat{f}})$$

where $\hat{h}$ is hit rate and $\hat{f}$ is false-alarm rate and $N$ is the number of to-be-remembered items. Pashler-K (rather than Cohen-K) is the recommended estimate for whole-display protocols such as the one used in this experiment^76^.

## Results

**Figure 2:**
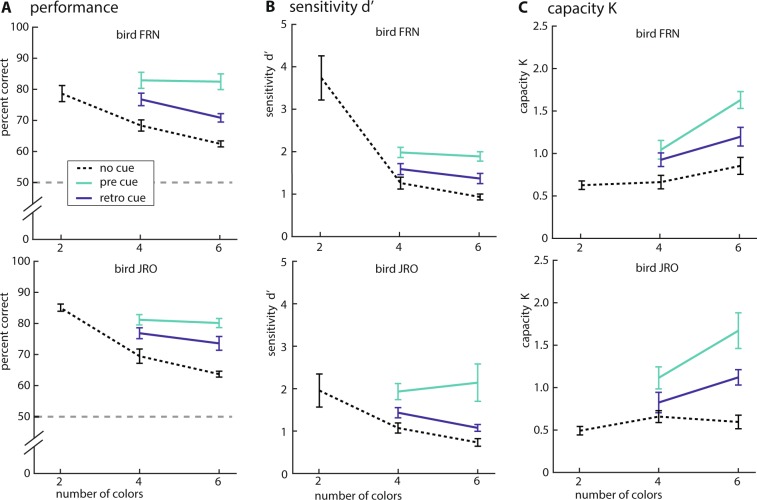
The effect of pre- and retro cues on performance. Both clues lead to an improvement in performance, visible in both animals and at all WM-loads. Error-bars indicate SEM, line style indicates the type of cue. (**A**) Behavioral performance (percent correct) of both crows (FRN, JRO) as a function of WM-load (2, 4, 6 colors) and cue-condition. Grey dashed line indicates chance-level. (**B**) Sensitivity (d’) of both crows as a function of WM-load and cue-condition. (**C**) Pashler’s K of both crows as a function of WM-load and cue-condition.

Both birds (FRN, JRO) performed 850 trials daily for 10 days with an overall performance that was well above chance (mean ± SD FRN: 76.54% ± 7.24, JRO: 74.15% ± 7.52). In the no-cue condition performance declined significantly with an increase in memory load (2, 4 and 6) (Fig. 2A) (one-way ANOVA FRN: F(2, 27) = 49.00, p < 0.001; JRO: F(2, 27) = 18.47, p < 0.001; post-hoc test: 2-colors FRN: M = 85.45 SD = 3.69 JRO: M = 77.30 SD = 8.05; 4-colors FRN: M = 69.77 SD = 7.29 JRO: M = 67.19 SD = 5.63; 6-colors FRN: M = 63.95 SD = 2.98 JRO: M = 61.36 SD = 2.99). This replicated the results of our previous study using a change-localization paradigm^70^.

A two-way ANOVA tested the effect of the cues and memory load on performance. Both birds benefited from the presence of cues: both pre and retro-cue significantly increased the performance compared to the no-cue condition (Fig. 2A; ANOVA FRN: F(2, 54)** = **31.71, p < 0.001; JRO: F(2, 54) = 38.71, p < 0.001; post-hoc test: no-cue: FRN M = 66.8 SD = 6.19; JRO M = 64.2 SD = 5.31; pre-cue FRN M = 81.0 SD = 4.88; JRO: M = 81.3 SD = 7.74; retro-cue FRN: M = 75.5 SD = 6.41; JRO M = 71.5 SD = 6.01) with a memory load effect (ANOVA FRN: F(1, 54) = 5.35, p = 0.03; JRO: F(1, 54) = 6.53, p = 0.01) but there was no interaction between both factors (ANOVA FRN: F(2,54) = 0.88, p = 0.42; JRO: F(2,54) = 0.28, p = 0.14).

Using a GLMM (binomial distribution, logit link function, bird as random variable) on the binary (correct/incorrect) raw-data we confirmed the significant effect of load on performance (estimate = −0.2, t(3872) = −8.09, p < 0.001). Using GLMM (binomial distribution, logit link function, bird as random variable) model comparisons we further demonstrated that a full model gave a significantly better fit than restricted models without the factors pre- (p < 0.01) or retro-cue (p < 0.01).

Along with the performance (percent correct), the d’ significantly decreased with the memory load in the no-cue condition (Fig. 2B; one-way ANOVA FRN: F(2, 27) = 23.30, p < 0.001; JRO F(2, 27) = 6.74, p < 0.001); post-hoc test: 2-colors FRN: M = 3.73 SD = 1.65 JRO: M = 1.95 SD = 1.23; 4-colors FRN: M = 1.26 SD = 0.47 JRO: M = 1.07 SD = 0.39; 6-colors FRN: M = 0.93 SD = 0.23 JRO: M = 0.73 SD = 0.29). Both birds were more accurate to detect the change in the cue conditions compared to the no-cue condition (Fig. 2B ANOVA FRN: F(2, 54) = 23.89, p < 0.001; JRO: F(2, 54) = 14.02, p < 0.001; post-hoc test: no-cue: FRN M = 1.06 SD = 0.39; JRO M = 0.90 SD = 0.38; pre-cue FRN M = 1.93 SD = 0.36; JRO M = 2.03 SD = 1.07; retro-cue FRN: M = 1.47 SD = 0.41; JRO M = 2.25 SD = 0.38) with a memory load effect only in FRN (ANOVA FRN: F(1, 54) = 4.69, p = 0.04; JRO: F(1, 54) = 0.83, p = 0.36) but there is no interaction between both factors (ANOVA FRN: F(2, 54) = 0.46, p = 0.63; JRO: F(2, 54) = 1.08, p = 0.34).

The capacity K did not differ between the no-cue conditions by the WM load (Fig. 2C; one-way ANOVA FRN: F(2, 27) = 2.65, p = 0.08; JRO: F(2, 27) = 1.40, p = 0.26). Both crows show a higher Pashler-K on the cue conditions when comparing with the no-cue condition, demonstrating an increase in information about the sample-array (Fig. 2C; ANOVA FRN: F(2, 54) = 24.92, p < 0.001; JRO: F(2, 54) = 18.58, p < 0.001; post-hoc test: no-cue: FRN M = 0.75 SD = 0.27; JRO M = 0.62 SD = 0.24; pre-cue FRN M = 1.46 SD = 0.39; JRO M = 1.39 SD = 0.61; retro-cue FRN: M = 1.06 SD = 0.35; JRO M = 0.97 SD = 0.34) with a memory load effect (ANOVA FRN: F(1, 54) = 12.48, p < 0.001; JRO: F(1, 54) = 6.44, p = 0.01), but there is no interaction between both factors (ANOVA FRN: F(2, 54) = 0.57, p < 0.57; JRO: F(2, 54) = 3.04, p = 0.06).

**Figure 3:**
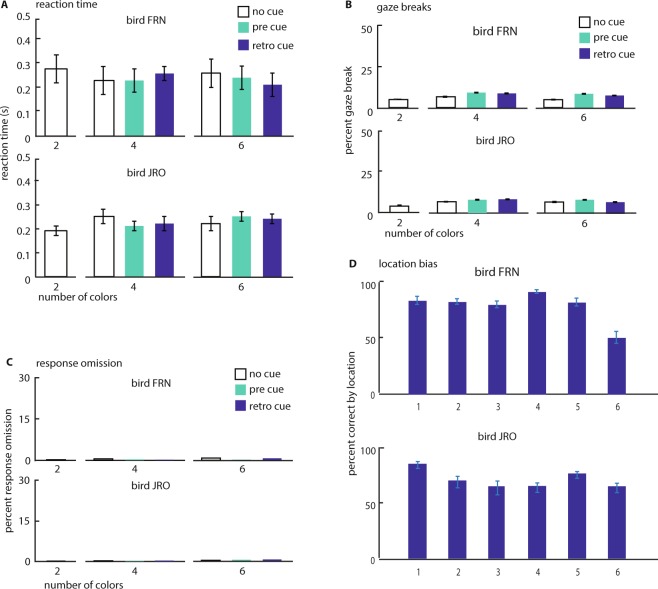
Reaction time and behavioral bias of both crows as a function of WM-load and cue-condition. Error-bars are SEM. (**A**) Reaction time on correct trials is not systematically affected by WM-load or cue-condition. (**B**) Percent trials with of gaze-breaks (head-movement during the trial) is slightly increased on cued trials. (**C**) Percent trials with response-omission (no response at the end of the trial) is unaffected by task-condition. (**D**) Percent correct as a function of stimulus location. Bird FRN has a lower performance on one location.

To determine whether there were any spatial response biases, we analyzed the performance by location. JRO showed a higher percent correct on location 1 while FRN, instead, showed a significantly lower performance in location 6 (Fig. 3D; ANOVA FRN: F(5, 54) = 16.86, p < 0.001 and post-hoc: location 6: M = 51.75 SD = 17.34; JRO: F(5, 54) = 3.51, p = 0.008 post-hoc: location 1: M = 88.02 SD = 9.95). Since the stimuli were presented randomly at six locations, this does, however, not explain the effects of pre- or retro-cues.

The reaction times increased on incorrect compared to correct choices, but only for FRN (one-way ANOVA FRN: F(1,18) = 6.98, p < 0.001; post-hoc test FRN: corr: M = 0.36 s SD = 0.07; incorr: M = 0.39 s SD = 0.16; JRO: F(1,18) = 2.61, p = 0.12). Both birds had a similar reaction time on correct responses across conditions (Fig. 3A; ANOVA FRN: F(2, 54) = 0.02, p = 0.98; JRO: F(2, 54) = 0.01, p = 0.99) and with increasing number of items to remember (ANOVA FRN: F(2, 54) = 0.04, p = 0.83; JRO: F(2, 54) = 0.29, p = 0.58: post-hoc test: 4-colors FRN: 0.30 s SD = 0.19; JRO M = 0.31 s SD = 0.09; 6-colors FRN: M = 0.39 s SD = 0.19; JRO M = 0.34 s SD = 0.1) and no interaction between the 2 factors (ANOVA FRN: F(2, 54) = 0.29, p = 0.74; JRO: F(2, 54) = 0.87, p < 0.42).

Comparing at each memory load, the gaze-breaks rate in no-cue trial was significantly lower (Fig. 3B; ANOVA FRN: F(2, 30) = 34.85, p < 0.001; JRO: F(2, 30) = 13.12, p < 0.001; post-hoc test: FRN: M = 11.49% SD = 2.50; JRO M = 13.74% SD = 1.64). The omission rate (the percentage of no response trials) at the choice was extremely low, covering only the 0.31% in FRN and the 0.25% in JRO of the total responses (Fig. 3C), underlining the full engagement of the crow at each trial.

## Discussion

In this study, we tested if crows can utilize spatial cues to optimize their WM capacity either before or after the encoding of the stimulus-material. We found that crows, like humans and monkeys, can use such cues to direct attention and select specific visual stimuli for WM maintenance (pre-cue). Importantly, we also demonstrate that crows can control their WM during stimulus-maintenance (retro-cue). In this case the animals were able to optimize their memory capacity even though the stimuli were no longer visible on the screen and already held in WM. These results demonstrate two distinct mechanisms that the crows use to tightly control WM which enables them to make the most of this “bottleneck of cognition.” It is important to note that this does not signify an increase in the capacity of WM but rather the economical use of this resource through different effective control mechanisms.

The behavioral protocol used in this study was modified from a previous experiment. Before, we used a change-localization protocol to assess the WM capacity of crows and to compare it to the capacity of monkeys^70^. Here, we used a change-detection protocol to maintain chance-level consistent at 50% across all WM-loads (2, 4 and 6 colors). As in the previous study, we found that an increase in the number of colors led to a decrease in performance, supporting our previous interpretation that the reported performance-decrement was the result of an increased WM-load. In the previous study, we estimated the capacity-limit at about four items^70^. With the new change-detection protocol, capacity K seems to plateau only in one animal (JRO) at this load (Fig. 2C). In the other animal (FRN) this limitation is not evident. This improved performance could reflect that the new paradigm (change detection) is easier than the old paradigm (change localization). Or, more likely, the continued training after our initial tests gave rise to performance benefits. However, since this improvement is evident only in one animal it should not receive too much attention.

To provide a complete overview over the data, we included a range of common but inconsistently used quantifications of WM performance (percent correct as the most direct and intuitive approach, sensitivity (d’) to control for shifts in response-biases^77^, Pashler’s capacity K to quantify WM capacity^76^. All of these quantifications revealed significant WM improvements on cued trials. Especially the pre-cue resulted in large improvement in performance that is consistent with previous results in humans^16,22,78^. This high effectivity of the pre-cue can be attributed to attention. By attending only to relevant information and thereby selecting only cued stimuli for maintenance the animals are able to greatly optimize their WM capacity. This is most evident in the fact that the performance on pre-cue trials did not show an effect of WM-load but was indistinguishable between 4- and 6-color trials. While attention has already been demonstrated in birds^39,41^, to our knowledge it has never been shown before that birds can use attention to maximize WM capacity.

Importantly, the crows were also able to use retro-cues to optimize WM capacity. In this case the underlying mechanism is not as clear as in the case of the pre-cue. At the time of the retro-cue presentation the crows already had to hold the stimulus-array in WM. Consequentially, WM capacity was already taxed and the animals could not simply rely on attentional selection to encode only relevant stimuli. Notably, the effect we observed in the crows is comparable to human data^16,22,25–33,77,79^.

A critical control in the experiments was possible by utilizing camera-based head-tracking. By training the animals to hold the head still and straight throughout each trial we could preclude the use of simple, non-cognitive strategies - for instance rotating the beak towards a cued location. Only with this control is it possible to conclude that the observed improvement in performance is due to attentional and executive mechanisms. We did find a small but significant increase in the number of gaze-breaks on cued as compared with no-cue trials (Fig. 3). This increase is likely due to the fact that the animals had to actively inhibit directing their gaze towards the cue stimuli. While the number of response-omissions was near zero on all conditions we found an increase in reaction time on incorrect responses (significant only in bird FRN) likely indicating greater uncertainty in the animals. Reaction time was otherwise unaffected by the different aspects of the paradigm (load, cue-condition) indicating the consistently high motivation of the animals.

The crows benefitted from retro-cues at loads of 4 and 6 items. In contrast, the single study in rhesus macaques found that the animals only benefitted from retro-cues on the low WM-load of two stimuli while they had no advantage at the higher load of three stimuli^56^. The rhesus monkey study also reported an improvement in reaction time that we could not replicate in the crows irrespective of the memory load. The reason for this is probably a difference in methodology; the crows had to withhold the response for 400 ms and were therefore unable to respond as soon as they made their decision. While it is difficult to say with certainty if the performance differences between crows and monkeys reflect true species-differences or merely the particularities of the experiments it does highlight the high level of executive control exerted by the crows. This is an important notion since it stresses that the cognitive system the crows used in this experiment is indeed WM as opposed to other forms of visual short-term memory that do not have an executive control component. This distinction is often not made clear in animal experiments even though the systems of WM and short term memory^80^ imply rather different cognitive complexity.

A variety of mechanistic explanations accounting for the enhancement effect of the retro-cue has been discussed in the human literature. Souza and Oberauer^77^ provide a comprehensive overview of these hypotheses. Briefly, the ideas evolve around three principles: The improvement of memory-maintenance (protection from decay, protection from interference or attentional refreshing); The removal of irrelevant stimuli, much like proposed in the directed forgetting literature^51,57,58^; Improvements at the time of decision (retrieval head-start, prioritization for retrieval). These mechanisms could also interact, for example it is possible that the maintenance of a cued item is strengthened while non-cued items are simultaneously forgotten^77,79,81^. Since the current experiment cannot discriminate between these explanations, further investigation is needed to provide a mechanistic understanding. Nevertheless, all proposed mechanisms imply some form of executive control over WM. Therefore, our data not only demonstrates that crows use attention to control stimulus-selection for WM it also demonstrates executive control over WM.

The presence of executive control in the evolutionary distant lines of birds and mammals could indicate one of two things. One possibility is that WM and its control mechanisms are evolutionary ancient traits that are likely shared by most (if not all) amniotes. Alternatively, these traits are the result of convergent (or parallel) evolution where a similar evolutionary pressure to develop cognitive abilities gave rise to a comparable cognitive toolbox. The case of evolutionary convergence seems to be more likely since executive control over WM was not demonstrated in a broad range of species. Admittedly, this might be due to the lack of systematic evaluation across amniotes. Regardless of the evolutionary model, our data shows that executive control is not a specific human or even a primate facility but that it is a core component of complex cognition that can be found also in our very distant relatives.

## Supplementary information

Supplementary Material.

## Data Availability

The datasets generated during the current study are available from the corresponding author on reasonable request.
