# Supplementary material for: Crows control working memory before and after stimulus encoding
Source: Sci Rep. 2020 Feb 24;10:3253. doi: 10.1038/s41598-020-59975-4 (PMC7039964; doi:10.1038/s41598-020-59975-4)
Supplement: Supplementary file 1 — Supplementary Material. [file 41598_2020_59975_MOESM1_ESM.docx]

**Crows control working memory before and after stimulus encoding.**

**Erica Fongaro**, **Jonas Rose***

Avian Cognitive Neuroscience, Institute for Cognitive Neuroscience, Ruhr-Universität Bochum, 44801 Bochum, Germany.

*****corresponding author

E-mail (corresponding author): [jonas.rose@rub.de](mailto:jonas.rose@rub.de)

Supplementary Materials

| 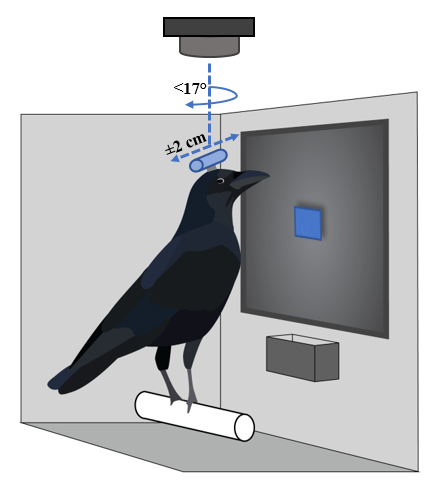 |
| --- |

**Fig. S1. The head-tracking system.** The bird was placed on a wooden perch in front of the touchscreen such that the maximum distance from the bird’s eye to the screen was 7 cm. The animal’s head position and rotation were tracked in the horizontal plane, using a computer-vision camera. For head-tracking, a custom 3d-printed reflector was mounted on a surgically implanted light-weight custom head-post and removed after each experimental session. The stimulus location is visible only in their monocular vision.

**Supplementary Note 1.**

Since the stimuli were presented only in the monocular fields, we tested if the birds performed better in one of the two hemispheres. We run a t-test for each condition comparing left and right performances (FRN: no-cue: right M = 79.95 % SD = 3.59; left M = 80.86 % SD = 2.96; pre-cue: right M = 84.68 % SD = 2.75; left M = 89.86 % SD = 1.69; retro-cue: right M = 84.09 % SD = 2.01; left right M = 80.33 % SD = 2.44; JRO no-cue right M = 68.29 % SD = 2.69; left M = 54.95 % SD = 5.42; pre-cue: right M = 77.97 % SD = 2.50; left M = 73.24 % SD = 3.48; retro-cue: right M = 70.23 % SD = 2.59; left M = 63.28 % SD = 3.24) (t-test FRN: no-cue : t(19) = -0.34, p = .73; pre-cue: t(19) = 3.01, p < .01; retro-cue: t(19) = 2.68, p = .014; JRO: no-cue: t(19) = -2.98, p < .01; pre-cue: t(19) = -2.34, p = .029; retro-cue: t(19) = -2.53, p < .002).

Overall, the birds had an opposite lateralization effect. While FRN showed a significant left screen preference only when involving cues, JRO maintained the right screen preference also during no-cue trials. Due to the divergent results and the small number of subjects, we cannot directly link the presence of a lateralization effect in corvids with the cue-effect on this WM task. Accordingly, we interpret the data as an arbitrary preference of the bird for one side of the screen preserved at each condition, rather than a laterality in visual attention triggered by cued stimuli.
